# Correlative Changes in Endogenous Polyamines and Hormones Associated with Aging in Ancient *Cinnamomum camphora*

**DOI:** 10.3390/plants15111752

**Published:** 2026-06-04

**Authors:** Jinling Feng, Mengping He, Jindian Sun, Xinyu Wen, Guanrong Ye, Yangyang Feng, Qingshan Chen, Hongwei Wu, Yousry A. El-Kassaby, Zhijian Yang

**Affiliations:** 1 College of Forestry, Fujian Agriculture and Forestry University, Fuzhou 350002, China; fengjinling@fafu.edu.cn (J.F.); hemengping@fafu.edu.cn (M.H.); sunjindian@foxmail.com (J.S.); wxiny@fafu.edu.cn (X.W.); yeguanrong@fafu.edu.cn (G.Y.); fengyangyang@fafu.edu.cn (Y.F.); chenqingshan@fafu.edu.cn (Q.C.); wuhongwei@fafu.edu.cn (H.W.); 2 Department of Forest and Conservation Sciences, Faculty of Forestry, University of British Columbia, 2424 Main Mall, Vancouver, BC V6T 1Z4, Canada; 3 Ministry of Education International Joint Laboratory on Genetic Improvement of Multiple Cropping Crops, Jiangxi Agricultural University, Nanchang 330045, China

**Keywords:** ancient tree, aging, senescence, polyamines, endogenous hormones, *Cinnamomum camphor*, hormone balance, age prediction

## Abstract

Plant aging and senescence are key determinants of lifespan, yet the coordinated changes in endogenous polyamines and hormones during long-lived tree aging remain largely unclear. *Cinnamomum camphora* exhibits sequential senescence from leaves to trunk, with leaf physiology shifting toward senescence around 450 years. This study aimed to clarify the patterns of polyamines and hormones across a wide age gradient (10–810 years) and their associations with aging in ancient *C. camphora*. Newly expanded leaves were analyzed using curve fitting, correlation, regression, and path analysis. Tree age significantly influenced most polyamine and hormone indices, except for indole-3-acetic acid (IAA), abscisic acid (ABA), and salicylate glucoside (SAG). Spermidine (Spd) and gibberellic acid (GA) were negatively correlated with aging, and cytokinin (CK) and cadaverine (Cad) were positively correlated with aging. Free salicylic acid (SAF) was closely related to the senescence transition point. Polyamines and hormones interacted strongly. Cad was positively correlated with CK, and Spd was positively correlated with GA. A model combining Spd, GA/ABA, and CK/GA reliably predicted ancient tree age. Overall, Spd and CK exhibited the strongest negative and positive correlations with aging, respectively, providing insights into the physiological regulation of longevity in ancient trees.

## 1. Introduction

Aging and death are inevitable outcomes for plants, influenced by both genetic and external factors [1,2]. As plants age, they undergo gradual physiological changes [3]. This process involves slow, cumulative alterations that accumulate over development [4]. Senescence represents the final stage of aging, characterized by gradual functional decline or death of plant organs or tissues [5,6]. Therefore, aging and senescence collectively contribute to the lifespan of plants. These processes can be observed at three levels: the cell, tissue, and whole plant [7]. While recent research has predominantly focused on cellular and tissue-level aging and senescence, further insights are still needed to fully understand plant longevity [8,9].

Plant endogenous hormones and polyamines are widely reported to correlate with aging and senescence in cells and tissues [10,11]. Endogenous hormones in plants, such as indole-3-acetic acid (IAA), cytokinin (CK), gibberellin acid (GA), abscisic acid (ABA), and salicylic acid (SA), are synthesized within the plant and show strong physiological potential. Small changes in their levels and balance can be related to leaf or organ aging [12,13,14]. Research has shown that SA and ABA can be associated with accelerated aging [14,15], while CK, IAA, and GA can be associated with delayed aging [16,17]. However, some studies suggest that ABA and GA may not be clearly linked to aging [18,19]. Additionally, SA has been found to be associated with senescence in leaves but with delayed senescence in fruit organs [14,20]. Recent studies indicate that the ratios of IAA, ABA, GA, and CK are more strongly related to aging and senescence than individual hormones levels [12].

Polyamines (PAs), which are low-molecular-weight aliphatic nitrogenous bases with strong physiological activity, are related to cell growth, differentiation, proliferation, and senescence [21]. Common PAs include putrescine (Put), spermidine (Spd), spermine (Spm), and cadaverine (Cad) [22]. Different types of PAs show varying patterns with organ and cell aging and senescence [11,23]. Some studies have shown that endogenous PA levels in plant tissues decrease with organ age [24,25,26], while others have observed an initial increase followed by a decrease in PA content [27]. In summary, while progress has been made in understanding relationships between plant endogenous hormones, polyamines, and aging, controversy remains regarding their directional correlations with aging and senescence in cells and tissues. Moreover, whether endogenous hormones and PAs are more strongly affected by age or by the environment, and how their interactions correlate with aging and senescence, require further investigation.

Previous studies on plant aging and senescence related to endogenous hormones and PAs have predominantly focused on annual or perennial herbs, with relatively few investigations into perennial woody plants [28,29]. Ancient trees, generally defined as those over 100 years old [30], undergo a gradual process of aging and senescence [31]. Research into the aging and senescence of ancient trees not only helps us understand patterns related to longevity but also aids in rejuvenating these trees [11]. Timely detection of physiological senescence in ancient trees and implementing appropriate rejuvenation measures can help reduce their decline and death, thereby preserving historical landscapes [32,33]. In the Sanming area of China, within a 12 km radius, *Cinnamomum camphora* trees ranging from 100 to 800 years old have been preserved. This provides a unique opportunity to study plant longevity by examining the aging and senescence in perennial woody plants under relatively consistent environmental conditions. According to previous research, *C. camphora* undergoes programmed senescence from outside to inside, in the order of leaves, branches to the center growth (trunk). The center growth (crown width and diameter at breast height) of *C. camphora* remains vigorous growth within 0–800 years, but leaf physiological metabolism shifts toward senescence at 450 years [34]. Therefore, the leaves of ancient *C. camphora* trees can serve as an ideal model for researching this topic.

To better understand patterns related to plant longevity, it is important to explore relationships between aging and endogenous hormones and PAs. This study focused on the new leaves of ancient *C. camphora* trees, analyzing changes in endogenous PAs and hormone indices across ages ranging from 10 to 810 years. Using curve fitting, regression, and correlation analysis, we identified indices correlated aging and senescence, evaluated their statistical relationships, and developed a method to predict the age of ancient trees. By employing correlation and path analyses, we investigated the interrelationships between endogenous hormones and PAs to characterize patterns underlying the longevity of ancient trees.

## 2. Results

### 2.1. Tree Age and Endogenous Polyamine Content

**Figure 1:**
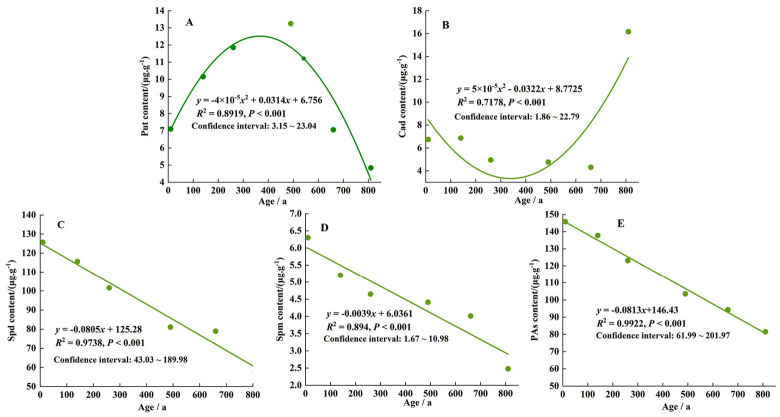
Curve fittings of tree age and endogenous polyamine indexes in *Cinnamomum camphora*. (**A**) Put content; (**B**) Cad content; (**C**) Spd content; (**D**) Spm content; (**E**) PAs content. To better understand the change trend of endogenous polyamine indexes with age, trend lines and fitting models were obtained. Age stage of 10, *n* = 6; age stage of 150, *n* = 6; age stage of 250, *n* = 3; age stage of 500, *n* = 3; age stage of 650, *n* = 1; age stage of 800, *n* = 1. The *R*^2^ value indicates the closeness of the linear relationship between endogenous polyamines and age. The *p* is used to test whether the trend is significant. *p* < 0.001 indicates extremely significant. The age in the *X*-axis labels represents the age of *C. camphor* tree. Put, Cad, Spd, Spm, and PAs in the *Y*-axis labels represent putrescine, cadaverine, spermidine, spermine, and total polyamines, respectively. The single data point of 810-year-old tree in (B) may be a potential outlier due to only one replicate in this age class; exclusion of this point would significantly reduce the *R*^2^ value and alter the linear fitting trend.

Contents of putrescine (Put), cadaverine (Cad), spermidine (Spd), spermine (Spm), and total polyamines (PAs) were significantly affected by the age of *C. camphora* trees (Figure 1). Curve fitting between polyamine contents and tree age yielded *R*^2^ > 0.71. Descending order of *R*^2^ were PAs, Spd, Spm, Put, and Cad. Contents of Put and Cad followed binomial models, while PAs, Spd and Spm followed linear models. As tree age increased, Put content initially increased and peaked at approximately 350 years, respectively. Meanwhile, contents of PAs, Spm and Spd decreased with tree age.

### 2.2. Tree Age and Endogenous Hormone Content

**Figure 2:**
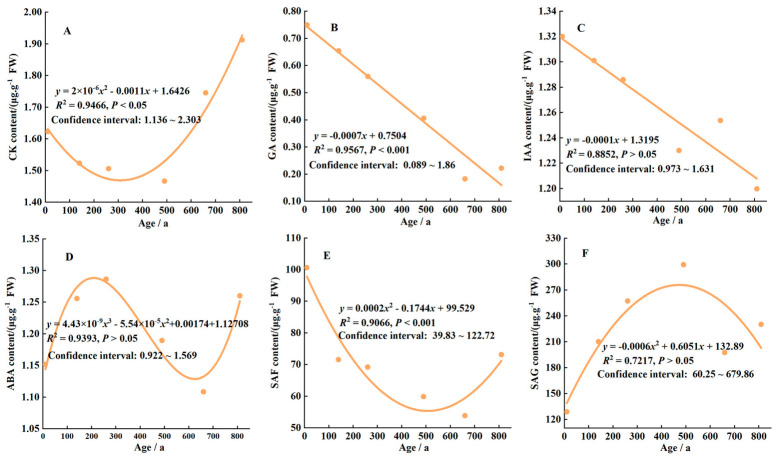
Curve fittings of tree age and endogenous hormone content in *C. camphor*. (**A**) CK content; (**B**) GA content; (**C**) IAA content; (**D**) ABA content; (**E**) SAF content; (**F**) SAG content. To better understand the change trend of endogenous hormones content with age, trend lines and fitting models were obtained. Age stage of 10, *n* = 6; age stage of 150, *n* = 6; age stage of 250, *n* = 3; age stage of 500, *n* = 3; age stage of 650, *n* = 1; age stage of 800, *n* = 1. The *R*^2^ value indicates the closeness of the linear relationship between endogenous hormone content and age. The *p* is used to test whether the trend is significant. *p* > 0.05 indicates no significant. *p* < 0.05 indicates significant. *p* < 0.01 indicates extremely significant. The age in the *X*-axis labels represents the age of *C. camphor* tree; CK, GA, IAA, ABA, SAF, and SAG in the *Y*-axis labels represent cytokinins, gibberellin, auxin, abscisic acid, free salicylic acid, and bound salicylic acid, respectively, the same as Figure 3.

Contents of gibberellin (GA) and free salicylic acid (SAF) were extremely significantly affected by the tree age, while cytokinins (CK) content was significantly affected. In contrast, contents of indole-3-acetic acid (IAA), abscisic acid (ABA), and bound salicylic acid (SAG) were not significantly affected (Figure 2). Curve fitting between hormone contents and tree age yielded *R*^2^ > 0.72. Descending order of *R*^2^ was GA, CK, ABA, SAF, IAA, and SAG. Contents of CK, SAF, and SAG followed binomial models, GA and IAA followed linear models, and ABA content followed a piecewise plateau curve model. As tree age increased, CK and SAF contents first decreased and then increased, reaching minima at approximately 350 and 450 years, respectively. SAG content increased initially and then decreased, peaking at around 450 years. GA and IAA contents both decreased with increasing tree age. ABA content increased initially, then decreased, and increased again, reaching its highest point at about 200 years and lowest at about 600 years.

### 2.3. Tree Age and Endogenous Hormone Ratios

**Figure 3:**
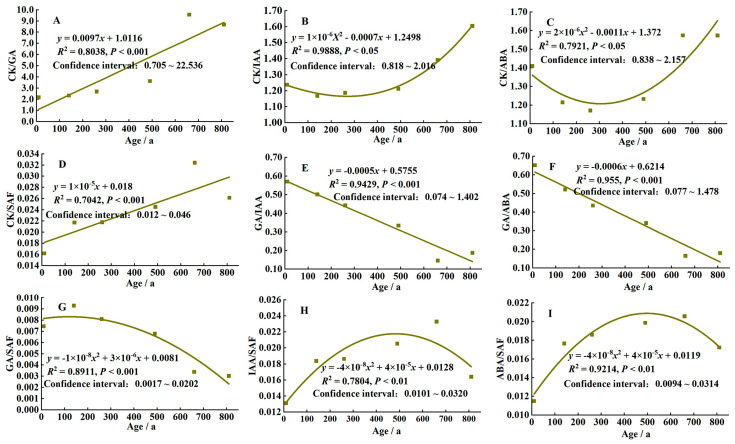
Curve fittings of tree age and endogenous hormone ratio in *C. camphor*. (**A**) Ratio CK/GA; (**B**) Ratio CK/IAA; (**C**) Ratio CK/ABA; (**D**) Ratio CK/SAF; (**E**) Ratio GA/IAA; (**F**) Ratio GA/ABA; (**G**) Ratio GA/SAF; (**H**) Ratio IAA/SAF; (**I**) Ratio ABA/SAF. To better understand the change trend of endogenous hormone ratios with age, trend lines and fitting models were obtained. Age stage of 10, *n* = 6; age stage of 150, *n* = 6; age stage of 250, *n* = 3; age stage of 500, *n* = 3; age stage of 650, *n* = 1; age stage of 800, *n* = 1. The *R*^2^ value indicates the closeness of the linear relationship between endogenous hormone ratio and age. The *p* is used to test whether the trend is significant. *p* > 0.05 indicates not significant. *p* < 0.05 indicates significant. *p* < 0.01 indicates extremely significant.

Tree age significantly affected the ratios CK/GA, CK/SAF, GA/IAA, GA/ABA, GA/SAF, IAA/SAF, and ABA/SAF, and also significantly affected CK/IAA, and CK/ABA ratios (Figure 3). Curve fitting between hormone ratios and the age yielded *R*^2^ > 0.70. Descending order of *R*^2^ was CK/IAA, GA/ABA, GA/IAA, ABA/SAF, GA/SAF, CK/GA, CK/ABA, IAA/SAF, and CK/SAF. Ratio CK/GA, CK/SAF, GA/IAA, GA/ABA and GA/SAF fit linear models, while CK/IAA, CK/ABA, IAA/SAF, and ABA/SAF fit binomial models. As tree age increased, ratios CK/GA and CK/SAF increased, whereas ratios GA/IAA, GA/ABA, and GA/SAF decreased. Ratios CK/IAA and CK/ABA first decreased and then increased, reaching their lowest point around 350 years. In contrast, ratios IAA/SAF and ABA/SAF increased initially and then decreased, peaking around 450 years.

### 2.4. Age and Environmental Effects

Age-associated effect was greater than the environmental effect for all polyamines contents. Average age-associated effect value was 0.96; the three strongest age-related indices were Cad, Spd, and PAs contents (Table 1).

Average age-associated effect of endogenous hormones indices was 0.73, and age-associated effects were stronger for hormone ratios than for individual contents. For IAA and SAG contents, environmental effects were greater than the age-associated effects. However, for Contents of CK, GA, ABA, and SAF, as well as all measured hormone ratios, age-associated effects were greater than the environmental effects (Table 2).

### 2.5. Path Analysis of Aging-Related Indices

Path analysis with tree age as the dependent variable and polyamine and hormone contents as independent variables showed that total coefficients of CK and Cad were positive, with CK > Cad content, indicating that CK and Cad contents were positively associated with aging. However, the direct coefficient of CK was near zero, indicating that CK positively associated with aging mainly through indirect effects, while its direct effect was negligible. Total coefficients of Put, Spd, Spm, GA, IAA, and SAF contents were negative, in absolute order with Spd > GA > Spm > SAF > Put > IAA (Table 3). This indicates that these six indices were negatively associated with aging, with Spd showing the strongest negative correlation, followed by GA, Spm, SAF, Put, and IAA. The total coefficient of ABA content was near zero, indicating little correlation between ABA and aging.

### 2.6. Correlation Analysis

**Figure 4:**
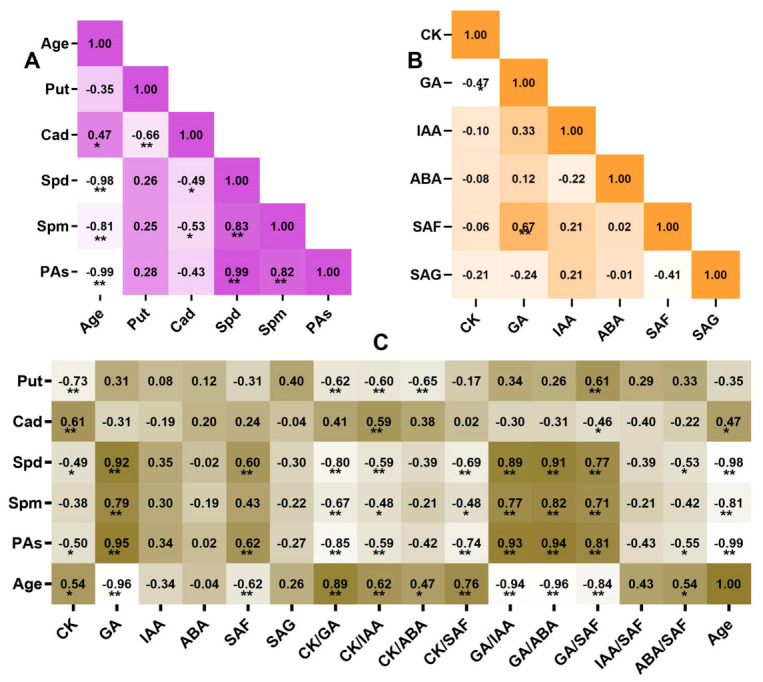
Correlation analysis of endogenous polyamines and hormones indexes on the aging of *C. camphor*. (**A**) Correlations among endogenous polyamine contents; (**B**) Correlations among endogenous hormone contents; (**C**) Correlations among endogenous polyamines, hormones, and tree age. Note: *, *p* < 0.05; **, *p* < 0.01. Put, Cad, Spd, Spm, PAs, CK, GA, IAA, ABA, SAF, and SAG represent putrescine, cadaverine, spermidine, spermine, total polyamines, cytokinins, gibberellin, auxin, abscisic acid, free salicylic acid, and bound salicylic acid, respectively.

Correlation among polyamines showed that Put content was significantly negatively correlated with Cad content. Cad content was significantly negatively correlated with Spd and Spm contents. Spd content was strongly positively correlated with Spm and PAs contents. Spm content was strongly positively correlated with PAs content (Figure 4A). For hormones, CK content was negatively correlated with GA content. GA content was strongly positively correlated with SAF content (Figure 4B).

Tree age showed a strong positive correlation with CK/GA, CK/IAA, CK/SAF ratio, CK content, CK/ABA, and ABA/SAF ratio. Conversely, tree age was strongly negatively correlated with GA and SAF content, GA/IAA, GA/ABA, and GA/SAF ratio. Tree age was positively correlated with Cad content and negatively correlated with Spd, Spm, and PAs content (Figure 4C).

CK content was highly significantly positively correlated with Cad content, and highly significantly or significantly negatively correlated with Put, Spd, and PAs content. GA content showed a highly significant positive correlation with Spd, Spm, and PAs content. SAF content was highly significantly positively correlated with Spd and PAs content. The CK/GA ratio was highly significantly negatively correlated with Put, Spd, Spm, and PAs content. The CK/IAA ratio was highly significantly positively correlated with Cad content, and highly significantly or significantly correlated with Put, Spd, Spm, and PAs content. The CK/ABA ratio exhibited a highly significant negative correlation with Put content. The CK/SAF ratio was highly significantly or significantly negatively correlated with Spd, Spm, and PAs content. Both the GA/IAA and GA/ABA ratios were highly significantly positively correlated with Spd, Spm, and PAs content. The GA/SAF ratio was significantly negatively correlated with Cad content but highly significantly positively correlated with Put, Spd, Spm, and PAs content. Lastly, the ABA/SAF ratio was significantly negatively correlated with Spd and PAs content (Figure 4C).

### 2.7. Age Prediction Model

With tree age as the dependent variable *Y* (ranging from 10 to 810 years) and 20 polyamine and hormone indices as independent variables *X_i_*, stepwise regression analysis yielded the following model:

*Y* = 123.891 − 7.663*X*_3_ + 20.341*X*_13_ − 290.560*X*_18_, adjusted *R*^2^ = 0.987, *p* < 0.001 (1)

In this model (1), *X_3_* represents Spd content, *X*_13_ represents CK/GA, *X*_18_ represents GA/ABA). K-fold validation was performed, with a prediction error of 16.8 years, indicating high fitting accuracy but potential overfitting due to limited sample size. These results indicate that Spd content, CK/GA and GA/ABA ratio are key factors associated with tree age. The prediction model showed good fitting performance but should be used with caution due to overfitting risk. Independent validation is recommended for broader application.

## 3. Discussion

Aging and senescence in plants are natural developmental processes regulated by both environmental and internal factors [1,2,35]. Physiological aging refers to the gradual physiological decline of trees with age; senescence refers to the final functional degradation stage of organs; longevity markers are indices stably correlated with tree lifespan. As plants age, they exhibit progressive physiological changes leading to senescence in later life stages [3,5]. For all endogenous polyamine and most hormone indices in leaves of ancient *C. camphora* trees, age-associated effects were stronger than environmental effects, with fitting *R*^2^ > 0.70. This suggests that endogenous polyamines and hormones are potential correlative indicators for evaluating aging and senescence in *C. camphora*.

Plant endogenous hormones are closely related to growth and development and show strong correlations with tree aging and senescence [10,36]. In *C. camphora*, tree age showed a strong negative relationship with SAF content, consistent with previous reports linking SAF to delayed aging [14,37]. SAF content initially decreased and then increased with age, indicating that SAF is related not only to aging but also to the transition toward senescence. Tree age significantly affected GA content, which declined with age, consistent with reports linking GA to delayed aging [38]. CK content was significantly positively correlated with tree age; while this correlation indicates CK can serve as a descriptive indicator of senescence progression, it does not imply causal promotion of aging and senescence transitions. CK increase may be an adaptive physiological response of the plant to delay or mitigate aging and senescence, or a by-product of other concurrent metabolic processes mediated by other biochemical factors [39,40]. CK content also exhibited a pattern of initial decrease followed by increase, implying involvement in both aging and senescence transitions [41].

IAA and ABA contents showed not significant age-related trends in *C. camphora*, consistent with reports that these hormones alone are not reliable age markers [18,19]. Environmental effects exceeded age-associated effects for IAA content, which declined with age, suggesting a minor role related to aging that is environmentally modulated. ABA content was more strongly related to age than environment and showed a fluctuating pattern with age, indicating involvement in developmental transitions [40]. In summary, SAF and GA contents were negatively associated with aging, CK content was positively associated with aging, and SAF and ABA contents were related to transitions across aging stages.

Aging and senescence are more strongly related to hormone balance than to individual levels [20]. In *C. camphora*, age-associated effects on hormone ratios were stronger than on individual contents, indicating that aging is closely linked to hormonal balance. Tree age significantly affected multiple hormone ratios, indicating strong interactions among CK, IAA, ABA, GA, and SAF, with the exception of ABA and IAA. It is possible that the regulatory pathways of IAA and ABA are relatively independent in plant aging and senescence. With increasing tree age, CK/GA and CK/SAF ratios increased, while GA/IAA, GA/ABA, and GA/SAF ratios decreased. CK/IAA, CK/ABA, and ABA/SAF ratios showed nonlinear patterns related to age. All these ratios were significantly correlated with tree age, with age-associated effects exceeding environmental effects, suggesting that CK/GA and CK/SAF ratios are positively associated with aging, while GA/IAA, GA/ABA, and GA/SAF ratios are negatively associated with aging.

Senescence transition refers to the physiological shift point of leaves toward senescence. Among the hormone ratios influencing senescence transition, CK content had minimal significant correlations with IAA and ABA content, and ABA content showed little significant correlation with SAF content. Path analysis indicated that the indirect association of CK on aging via GA was substantial, and CK and GA contents were negatively correlated. This suggests that aging patterns are related to metabolic interactions between CK and GA, possibly involving competition for common precursors [42]. Increased CK accumulation may be related to higher oxidative status and accelerated aging [43,44]. GA/IAA, GA/ABA, and GA/SAF ratios were negatively associated with aging, with GA as a central hub correlated with IAA, ABA, and SAF. GA content was positively correlated with IAA and SAF contents and negatively correlated with SAG content, suggesting relationships consistent with coordinated growth and stress responses [45,46,47]. The absence of a strong negative correlation between GA and ABA contents suggests that their relationship is not based on mutual accumulation but on related physiological processes such as redox balance [48,49,50,51]. Among hormone ratios, CK/GA and GA/ABA showed the highest age-associated effects (0.99 and 0.96, respectively), indicating that aging in *C. camphora* is closely related to balance among CK, GA, and ABA.

Polyamines are closely associated with plant aging [11]. In *C. camphora*, age-associated effects exceeded environmental effects for all polyamine contents, indicating that polyamine metabolism is predominantly related to tree age. Aging was positively correlated with Cad content and negatively correlated with Spd, Spm, and PAs content, suggesting that Cad is associated with accelerated aging [52], while Spd, Spm, and PAs are associated with delayed aging [26,53,54]. Cad content was negatively correlated with Spd and Spm content, while Put was positively correlated with Spd and Spm. These patterns align with known polyamine biosynthesis pathways [55,56]. Path analysis revealed that Spd showed the strongest negative association with aging, followed by Spm and Put. As *C. camphora* trees aged, Spd content continuously decreased, indicating that Spd synthesis was crucial for delayed aging.

Polyamines interact with various hormones in plant tissues [57]. In *C. camphora*, polyamine levels were correlated with multiple hormones. GA was positively correlated with Spd and PAs accumulation, while CK was positively correlated with Cad. SAF was positively correlated with Spd and PAs, and ABA appeared to counteract this relationship. Thus, Spd and Cad levels are embedded in a correlative network involving GA, CK, SAF, and ABA. GA showed the strongest positive correlation with Spd, indicating that GA is closely related to Spd accumulation, while CK is closely related to Cad accumulation.

Based on the discussions above, the contents of Spd, SAF, and GA, as well as the ratios GA/IAA, GA/ABA, and GA/SAF, were associated with delayed aging in *C. camphora* plants. Conversely, Cad and CK contents, along with the CK/GA and CK/SAF ratios, were linked to aging. Among these indicators, Spd content, GA content, GA/ABA ratio, and CK/GA ratio showed high accuracy (*R*^2^ > 0.90) and significant age effects (>0.96). Therefore, Spd content, GA/ABA ratio, and CK/GA ratio are key markers for physiological aging in ancient trees, consistent with regression model results.

Cad, SAF, and ABA contents, as well as CK/IAA, CK/ABA, and ABA/SAF ratios, were related to senescence transition. SAF content and ABA/SAF ratio showed high fitting reliability (age effect > 0.80). SAF content decreased then increased with age, reaching a minimum at 450 years, while ABA/SAF ratio peaked at about 450 years. These patterns suggest that leaf senescence in *C. camphora* tends to initiate around 450 years, consistent with previous physiological studies [34]. Among aging and senescence markers, Spd is widely conserved across organisms including trees, fungi, nematodes, insects, and rodents [58,59,60,61], suggesting that Spd may represent a universal correlate of longevity across taxa.

However, this study has certain limitations. The number of repeated samples for different age groups is uneven and limited. For the age groups of 650 and 810, there is only one tree in each age group. Although this limitation can be understood due to the scarcity of ancient trees, it undermines the statistical reliability of the results.

## 4. Materials and Methods

### 4.1. Plant Materials

Sanming City, located in Fujian Province, China, lies in the subtropical zone and features primarily low mountains and hills (alt.: 150 m, 117°30′–117°47′ E, 26°14′–26°25′ N). The city experiences an average annual temperature of 19.5 °C. The hottest month sees an average of 29.5 °C, while the coldest month averages 7.1 °C, with an average daily temperature of 9.6 °C. The annual active accumulated temperature is 6550 °C, and frost-free period ranges from 272 to 328 days. Average annual precipitation is 1726 mm, while average annual evaporation is 1624 mm, and the average annual relative humidity is 79%. The predominant soil types are yellow soil and red soil.

According to the age of *C. camphora* trees recorded in The Records of Famous and Ancient Trees of Sanming City, test trees at six age stages were selected: 10, 150, 250, 500, 650, and 800 years. Based on actual availability, 1–6 replicate trees were sampled per age class: six trees at each of the first two age stages, three trees at the 250 years, three trees at the 500 years, and one tree at each of the last two age stages, totaling 20 sampled *C. camphora* trees (Table 4). The 650- and 810-year-old trees are single observational samples due to the scarcity of ancient trees. Sample trees were selected within an urban radius of 12 km in Sanming City, Fujian Province, China, to ensure relatively consistent site conditions under the same climatic background. In early June 2022, 15 shoots from the outer middle of the crown of each sampled tree were randomly selected at 120° intervals, ensuring they were robust and free of diseases and pests. From each shoot, one fully mature new leaf was randomly collected. Leaves were pooled within each individual tree (not across age classes), frozen in liquid nitrogen, and stored at −80 °C for the determination of endogenous PAs and hormones contents. Biological replicates in statistical analyses were individual trees, with 20 independent observations included in regression and path analyses.

### 4.2. Determination of PAs

Approximately 1.0 g of mixed leaves was homogenized with 3 mL of 0.5% perchloric acid in a pre-cooled mortar, placed on ice for 1 h, and then centrifuged at 12,000× *g* for 20 min at 4 °C. A 500 μL aliquot of the supernatant was mixed with 1 mL of 2 mol/L NaOH and 7 μL benzoyl chloride. This mixture was vortexed for 20 s and incubated for 30 min at 37 °C. Then, 2 mL of saturated NaCl and 2 mL ether were added and the mixture was centrifuged at 15,000× *g* for 5 min at room temperature. A 1 mL aliquot of the supernatant was transferred to a 10 mL centrifuge tube, concentrated using a nitrogen blower, and then redissolved in 100 μL 60% methanol. The solution was filtered through a 0.22 μm microporous filter membrane for measurement. Standard solutions of putrescine (Put), spermidine (Spd), spermine (Spm), and cadaverine (Cad) were prepared by dissolving 0.1 g of each standard (Sigma-Aldrich, St. Louis, MO, USA) in ultra-pure water to make 10 mg/mL stock solutions. For these, 20 μL aliquots were subjected to the benzoylation reaction mentioned above, and the resulting solutions were diluted with 60% methanol to prepare standards at 100, 50, 25, 10, 5, 2.5 and 1 μg/mL. The contents of Put, Cad, Spd and Spm were determined using a Waters High Performance Liquid Chromatograph (Waters600, Waters Corporation, Milford, MA, USA). Each sample (10 μL) was injected into a 4.6 mm × 250 mm reversed-phase C-18 column at a flow rate of 0.8 mL/min and a column temperature of 40 °C. Analysis was performed with a 40 min run time using a UV-visible detector (UA-2450, Shimadzu Corporation, Kyoto, Japan) set at 340 nm excitation and 515 nm emission. Standard curves were constructed by plotting standard concentration against peak area. Contents in samples were calculated from standard curves and peak areas. The total PAs content was calculated as the sum of Put, Cad, Spd, and Spm contents [55,62].

### 4.3. Determination of Hormones

Approximately 0.1 g of mixed leaves sample was ground in a mortar. Then, 1 mL of pre-cooled extraction solution (80% methanol containing 1% BHT antioxidant) was added. The mixture was soaked at 4 °C overnight and then centrifuged at 8000× *g* for 10 min to obtain the supernatant. The residue was extracted with 0.5 mL of extraction solution for 2 h, centrifuged again, and the supernatant was collected. The two supernatants were combined and concentrated using a nitrogen blower, with nitrogen at 40 °C until no organic phase was present. Next, 0.5 mL petroleum ether was added for decolorization, performed for three times at 60–90 °C. After discarding the upper ether phase, the solution was adjusted to pH 6.8, dried with nitrogen, and then 0.5 mL mobile phase (0.1% acetic acid water: methanol = 6:4) was added. The solution was vortexed and filtered through a 0.22 μm membrane before measurement. Standard solutions of auxin (IAA), cytokinin (CK), gibberellin acid (GA_3_), and abscisic acid (ABA) (Shanghai Zhenzhen Company, Shanghai, China) were prepared in methanol containing 0.1% formic acid, with concentrations of 0.1, 0.2, 0.5, 2, 5, 20, 50, 200 μg/mL. Contents of CK, GA_3_, IAA, ABA were determined using an Agilent high performance liquid chromatograph (Agilent 1100, Agilent Technologies, Inc., Santa Clara, CA, USA). Leaf samples and standard solutions (10 μL each) were analyzed using a Kromasil C18 reverse-phase column (250 mm × 4.6 mm × 5 μm, AkzoNobel, Bohus, Sweden) at a flow rate of 0.8 mL/min, a column temperature of 30°C, and run time of 40 min. Detection was performed at 254 nm with mobile phase of 0.1% acetic acid in water: methanol = 6:4. Standard curves were constructed and sample contents were calculated as above [63].

### 4.4. Determination of Salicylic Acid (SA)

Approximately 0.5 g of mixed leaf sample was weighed and ground in liquid nitrogen. Then, 1 mL 90% (*v*/*v*) methanol was added, and the mixture was vortexed and sonicated for 3 min. The sample was centrifuged at 10,000× *g* for 15 min to obtain the supernatant. The residue was extracted with 0.5 mL 100% methanol, and the process was repeated. The two supernatants were combined and dried under nitrogen. Next, 0.25 mL of 5% (*v*/*v*) trichloroacetic acid was added to dissolve the residues completely. The solution was extracted twice with 0.8 mL ethyl acetate: cyclohexane (1:1). The upper phase contained free salicylic acid (SAF), which was concentrated under nitrogen. The lower phase contained salicylate glucoside (SAG), which was acidified with 0.3 mL 8 M HCl and incubated in a water bath at 80 °C for 1 h to release SAF, then concentrated under nitrogen. Concentrated SAF and SAG were each dissolved in 600 μL mobile phase and filtered through a 0.22 μm filter for measurement. SA contents were determined using an Agilent high performance liquid chromatograph (Agilent 1100, Agilent Technologies Co., Ltd., Santa Clara, CA, USA). Standard solution of SA (Shanghai Zzbio Co., Ltd., Shanghai, China) was prepared at 0.1, 0.2, 0.5, 2, 5, 20, 50, and 200 μg/mL. Samples and standards were analyzed on a Kromasil C18 reverse-phase column (250 mm × 4.6 mm × 5 μm) at 0.8 mL/min, 30 °C, 40 min run time, excitation 305 nm, emission 407 nm. Mobile phase was 0.1% acetic acid water: methanol = 4:6. Standard curves were constructed and sample contents were calculated as above [64].

### 4.5. Data Analysis

A limited number of (*x_i_*, *y_i_*) measurement pairs were obtained through the experiment, and the approximate function was obtained using these data, where *x_i_* represents the age of the samples in the *i* age group, and *y_i_* represents the average value of an indicator in the *i* age group. Using the software Excel 2021, scatter plots were constructed with tree age as the *X*-axis and the average values of each polyamine and hormone index as the *Y*-axis. To better understand the trend of data changes with age, a trend line was added, and the goodness of fit for different fitting models was compared to select the best fitting model. This process yielded equations relating tree age to each polyamine and hormone index [65]. The average value of each polyamine and hormone index at a corresponding tree age is referred to as the phenotypic value (*P*). The phenotypic value of any quantitative trait is the result of the combined effect of age-associated (*G*) and environmental (*E*) factors.

*P* = *G* + *E* (2)

Through ANOVA, the age-associated variance (*VG*), environmental variance (*VE*), and phenotypic variance (*VP*) were obtained. The age-associated effect (*G*) and environment effect (*E*) of polyamine and hormone indexes were estimated as follows [66]:

*G* = *VG*/(*VG* + *VE*) (3)

*E* = *VE*/(*VG* + *VE*) (4)

In order to select the most important variables from a large number of available options and explore the statistical relationships among the variables, ANOVA, correlation analysis, stepwise regression and path analysis were performed for each polyamine and hormone index data by SPSS 19.0 software.

## 5. Conclusions

Contents of polyamines, GA, SAF, and CK, as well as multiple hormone ratios, were significantly correlated with the age of *C. camphora* trees. In contrast, contents of IAA, ABA, and SAG were not significantly age-related. Age-associated effects were relatively weak for IAA and SAG but strong for polyamines and hormone ratios. Overall, polyamines showed the strongest age dependence, followed by hormone ratios, then individual hormone contents. Spd, SAF, and GA contents, as well as GA/IAA, GA/ABA, and GA/SAF ratios, were negatively correlated with aging in *C. camphora*, with Spd and GA showing the strongest negative associations. Conversely, Cad and CK contents, as well as CK/GA and CK/SAF ratios, were positively correlated with aging, with CK as the strongest positive correlate. Cad, SAF, and ABA contents, together with CK/IAA, CK/ABA, and ABA/SAF ratios, were related to senescence transitions, with SAF as a key marker. Leaf physiology in ancient *C. camphora* tends to shift toward senescence around 450 years. Polyamines and hormones formed a coordinated regulatory network. Cad is strongly correlated with CK, and Spd is strongly correlated with GA. Stepwise regression confirmed that Spd content, GA/ABA ratio, and CK/GA ratio are potential correlative indicators for evaluating physiological status and predicting the age of ancient *C. camphora* trees. Given that Spd is ubiquitous in living organisms, its abundance may serve as a conserved correlate of longevity across diverse taxa. These findings deepen our understanding of the hormonal and polyamine-mediated regulation of aging in ancient trees and provide practical correlative indicators for the conservation and rejuvenation of long-lived woody species.

## Figures and Tables

**Table 1:** Age-associated and environmental effects of endogenous polyamine indexes in *Cinnamomum camphora*.

Index	Age-Associated Variation	Environment Variation	Total Variation	*p*	Age-Associated Effect	Environment Effect
Put content	157.92	8.63	166.55	<0.001	0.95	0.05
Cad content	299.97	4.22	304.19	<0.001	0.99	0.01
Spd content	10,107.08	191.00	10,298.08	<0.001	0.98	0.02
Spm content	24.71	3.25	27.96	<0.001	0.88	0.12
PAs content	9647.08	204.23	9851.31	<0.001	0.98	0.02
Average	-	-	-	-	0.96	0.04

Note: Unit of endogenous polyamine content is μg/g. Age-associated effect = Age-associated variation/Total variation; Environment effect = Environment variation/Total variation.

**Table 2:** Age-associated and environmental effects of endogenous hormone indexes in *C. camphor*.

Index	Age-Associated Variation	Environment Variation	Total Variation	*p*	Age-Associated Effect	Environment Effect
CK content	0.45	0.23	0.68	0.020	0.66	0.34
GA content	0.84	0.03	0.87	<0.001	0.97	0.03
IAA content	0.03	0.20	0.23	0.860	0.13	0.87
ABA content	0.07	0.05	0.12	0.510	0.58	0.42
SAF content	3895.79	208.81	4104.60	<0.001	0.95	0.05
SAG content	24,395.37	53,439.86	77,835.22	0.320	0.31	0.69
Average of individual hormones content	-	-	-	-	0.60	0.40
CK/GA	169.75	2.10	171.85	<0.001	0.99	0.01
CK/IAA	0.43	0.32	0.75	0.040	0.58	0.42
CK/ABA	0.50	0.40	0.90	0.490	0.56	0.44
CK/SAF	0.00044	0.00009	0.00053	<0.001	0.83	0.17
GA/IAA	0.44	0.03	0.47	<0.001	0.94	0.06
GA/ABA	0.56	0.02	0.58	<0.001	0.96	0.04
GA/SAF	0.00010	0.00001	0.00011	<0.001	0.90	0.10
IAA/SAF	0.00018	0.00014	0.00022	<0.001	0.76	0.24
ABA/SAF	0.00016	0.00005	0.00020	<0.001	0.81	0.19
Average of hormones ratios	-	-	-	-	0.81	0.19
Total average	-	-	-	-	0.73	0.27

Note: Unit of endogenous hormone content is μg/g. Age-associated effect = Age-associated variation/Total variation; Environment effect = Environment variation/Total variation.

**Table 3:** Pathway analysis of endogenous polyamine and hormone contents on the aging of *C. camphor*.

Factor	Direct Coefficient	Indirect Coefficient									Total Coefficient
		→Put	→Cad	→Spd	→Spm	→CK	→GA	→IAA	→ABA	→SAF	
Put	−0.104	-	−0.046	−0.144	0.014	0.004	−0.114	0.001	0.001	0.036	−0.353
Cad	0.070	0.069	-	0.279	−0.028	−0.003	0.113	−0.001	0.002	−0.027	0.473
Spd	−0.563	−0.027	−0.035	-	0.044	0.003	−0.333	0.002	0.000	−0.069	−0.977
Spm	0.053	−0.027	−0.037	−0.466	-	0.002	−0.286	0.002	−0.002	−0.049	−0.810
CK	−0.006	0.076	0.042	0.276	−0.020	-	0.170	−0.001	−0.001	0.007	0.544
GA	−0.363	−0.033	−0.022	−0.516	0.042	0.003	-	0.002	0.001	−0.078	−0.963
IAA	0.006	−0.008	−0.013	−0.197	0.016	0.001	−0.122	-	−0.003	−0.025	−0.345
ABA	0.011	−0.013	0.014	0.009	−0.010	0.000	−0.044	−0.001	-	−0.003	−0.036
SAF	−0.115	0.032	0.017	−0.336	0.023	0.000	−0.245	0.001	0.000	-	−0.622

Note: The direct coefficient represents the magnitude of the direct effect of the factor on aging. The indirect coefficient indicates the magnitude of how a factor indirectly affects aging through other factors. The Total coefficient represents the total magnitude of the effect of the factor on aging, i.e., direct coefficient + sum of indirect coefficient via other factors. The arrow (→) represents the indirect coefficient from the row factor to the column factor through the corresponding intermediate variable pathway.

**Table 4:** Age and location of the 20 studied *C. camphora* trees.

Ancient Tree Code	Age Stage (a)	Age (a)	Location
-	10	10	Liedong Village, Liedong Street
-		10	Liedong Village, Liedong Street
-		10	Liedong Village, Liedong Street
-		10	Liedong Village, Liedong Street
-		10	Liedong Village, Liedong Street
-		10	Liedong Village, Liedong Street
350402003002	150	133	Xubi Village, Xubi Street
350402003001		143	Bikou Village, Xubi Street
350402001006		140	Liedong Village, Liedong Street
350403101002		145	Yanqian Village, Yanqian Town
350403200007		147	Hospital of Integrated Traditional Chinese and Western Medicine
350402002011		147	Liexi Village, Liexi Street
350403200001	250	252	Taijiang Village, Chengdong Town
350402002005		260	Liexi Village, Liexi Street
350402100024		271	Chendun Village, Chenda Town
350403101042	500	490	Jikou Village, Yanqian Town
350403200001		523	Taijiang Village, Chengdong Town
3504032000014		502	Liexi Village, Liexi Street
350402100012	650	660	Chendun Village, Chenda Town
350402100013	800	810	Chendun Village, Chenda Town

Note: a: year(s). For both “Age Stage” and “Age” columns, “a” represents age in years.

## Data Availability

The original contributions presented in this study are included in the article. Further inquiries can be directed to the corresponding authors.

## Abbreviations

The following abbreviations are used in this manuscript:

PAs	Polyamines
Put	Putrescine
Cad	Cadaverine
Spd	Spermidine
Spm	Spermine
GA	Gibberellin
CK	Cytokinins
IAA	Auxin
ABA	Abscisic acid
SA	Salicylic acid
SAF	Free salicylic acid
SAG	Bound salicylic acid
